# Quantitative risk assessment model of the presence of porcine epidemic diarrhea and African swine fever viruses in spray-dried porcine plasma

**DOI:** 10.3389/fvets.2024.1371774

**Published:** 2024-06-12

**Authors:** Fernando Sampedro, Pedro E. Urriola, Jennifer L. G. van de Ligt, Declan C. Schroeder, Gerald C. Shurson

**Affiliations:** ^1^Division of Environmental Health Sciences, School of Public Health, University of Minnesota, Minneapolis, MN, United States; ^2^Department of Veterinary Population Medicine, College of Veterinary Medicine, University of Minnesota, Minneapolis, MN, United States; ^3^Department of Animal Science, College of Agricultural and Natural Resource Sciences, University of Minnesota, St. Paul, MN, United States

**Keywords:** African swine fever virus, performance objective, risk assessment, spray-dried porcine plasma, swine viruses, thermal inactivation

## Abstract

**Introduction:**

There are no microbiological regulatory limits for viruses in animal feed and feed ingredients.

**Methods:**

A performance objective (PO) was proposed in this study to manufacture a spray-dried porcine plasma (SDPP) batch absent of any infectious viral particles. The PO levels of −7.0, −7.2, and −7.3 log TCID_50_/g in SDPP were estimated for three batch sizes (10, 15, and 20 tons).

**Results and discussion:**

A baseline survey on the presence of porcine epidemic diarrhea virus (PEDV) in raw porcine plasma revealed a concentration of −1.0 ± 0.6 log TCID_50_/mL as calculated using a TCID_50_-qPCR derived standard curve. The mean African swine fever virus (ASFV) concentration in raw plasma was estimated to be 0.6 log HAD_50_/mL (0.1–1.4, 95% CI) during a pre-clinical scenario (collected from asymptomatic and undetected viremic pigs). Different processing scenarios (baseline: spray-drying + extended storage) and baseline + ultraviolet (UV) radiation were evaluated to meet the PO levels proposed in this study. The baseline and baseline + UV processing scenarios were >95 and 100% effective in achieving the PO for PEDV by using different batch sizes. For the ASFV in SDPP during a pre-clinical scenario, the PO compliance was 100% for all processing scenarios evaluated. Further research is needed to determine the underlying mechanisms of virus inactivation in feed storage to further advance the implementation of feed safety risk management efforts globally.

## Highlights

- The SDPP production process has been validated to inactivate PEDV and ASFV (8.4–11.1 mean log reduction).
- Under the current conditions, the model estimated that an inactivation level (log-kill) of 7.0, 7.2, and 7.3 log must be achieved to manufacture a 10-, 15-, and 20-tons batch size, respectively.
- Performance Objective compliance rates were >95% for the baseline SDPP production scenario and 100% for the baseline + UV processing scenario.
- Chemical and physical factors that contribute to the inactivation of various swine viruses in feed ingredients during storage need to be determined.

## Introduction

Spray-dried porcine plasma (SDPP) is an important highly digestible, functional feed ingredient obtained from the blood of healthy pigs that provides significant health benefits beyond the amino acids it provides to the diets of weaned pigs [(1), (2)]. The use of effective functional ingredients and nutrients in weaned pig diets is essential for supporting optimal pig health without the use of growth-promoting antibiotics and for combatting antimicrobial resistance (3). Furthermore, animal-derived proteins from SDPP and various by-products from the rendering industry (e.g., blood meal, meat, and bone meal) are concentrated, highly digestible sources of nitrogen and phosphorus, and have a much lower environmental footprint than corn, soybean meal, and other grain-based by-products used in swine diets (4). Therefore, a quantitative risk assessment of the presence of swine viruses in SDPP is needed to evaluate its potential disease transmission role in feed.

Viruses that affect pigs and may contaminate swine blood and blood products are numerous and vary from enveloped to non-enveloped, single, and double-stranded DNA and RNA viruses, and range in size from the smallest (i.e., Porcine circovirus 2; PCV-2), to the largest (i.e., African swine fever virus; ASFV) (5–7). The SDPP manufacturers use a series of processing steps aimed at inactivating viruses in plasma, including spray-drying, and extended storage, and they are also evaluating the use of ultraviolet light as additional control steps for virus inactivation (Personal Communication, North American Spray Dried Blood and Plasma Producers; NASDBPP). The use of different inactivation mechanisms (i.e., rapid dehydration, UV, low-moisture storage) allows adding the inactivation levels achieved by each single processing step to estimate the total virus inactivation level, known as hurdle technology (8).

Spray drying has been used since the 1970’s as an efficient drying method aimed at processing liquid concentrates into powders to provide extended shelf-life and retain the activity of bioactive components (9). The very rapid changes in moisture, temperature, and mass/volume of particles during drying (in a matter of seconds) increase microbial and virus inactivation, and depending on the process conditions, spray-drying can be used as a pasteurization-like process (10). The increased need for safe animal feeding programs has led the industry to seek reliable additional processing steps that can increase pathogen inactivation if contamination occurs. Ultraviolet light radiation is a processing technology aimed at reducing microbial and viral contamination in water and liquid foods. Most UV devices operate at 254 nm (UV-C), which is considered the “germicidal UV” (11). Viruses are inactivated by UV-C light caused by photochemical damage to their nucleic acids (DNA/RNA), and this damage is irreversible due to a lack of repair mechanisms which leave the organisms unable to perform vital cellular functions and, hence, prevent the virus from multiplying (12, 13).

According to the International Commission of Microbiological Specifications in Food (ICMSF), the maximum microbiological concentration of a hazard in a food product at a specified step in the food chain before consumption is known as the Performance Objective (PO) (14–16). The PO is related to the contamination of the raw material and inactivation achieved during individual or multiple control steps and it can also be applied to feed safety. However, a PO level related to the presence of swine viruses has not been established for any feed ingredient.

Quantitative microbial risk assessment (QMRA) is a field of study aimed at quantifying the risk of illness due to the exposure to a pathogen through the consumption of contaminated food or feed. QMRA models have been developed mainly targeting foodborne human pathogens in food, and the availability of quantitative models to estimate the exposure of animal viruses via feed are very limited (17). Therefore, the aim of this study was to propose a PO level in SDPP that is capable of achieving a virus-free batch involving potential contamination of swine viruses such as PEDV and ASFV in a preclinical scenario and develop a quantitative model to evaluate the performance of several processing strategies to achieve the proposed PO levels.

## Methodology

### Spray-dried porcine plasma manufacturing process

Information on SDPP manufacturing process was obtained from a literature review and consultation with industry experts (European Animal Protein Association, EAPA and NASDBPP) to determine the effect of UV, spray-drying and extended storage heat on the inactivation of PEDV and ASFV.

The process of SDPP manufacturing begins with the collection of blood from animals inspected for human consumption at the slaughterhouse, which is subsequently treated with an anticoagulant, chilled, and transported to industrial facilities in which blood is centrifuged to separate the red blood cells from the plasma fraction. Plasma is then concentrated either by membrane filtration or a vacuum evaporator to achieve between 22 and 30% solids content and spray-dried at high temperatures (80°C) to convert it into a powder. The dried plasma then undergoes a particle filter and European and North American SDPP producers include an additional safety step of storing at room temperature (20°C) for 2 weeks (10). The SDPP manufacturers use a series of processing steps that can be identified as critical control points (CCP) in a Hazard Analysis and Critical Control Points (HACCP) aimed at removing or inactivating viruses in blood products. In the production of SDPP, a generic HACCP plan includes two CCPs (Table 1): spray-drying (CCP1) and extended storage at 20°C (CCP2). In addition, ultraviolet light (UV, CCP3) is currently being implemented in some European and US factories for virus inactivation (Personal communication, NASDBPP).

**Table 1:** Operational range of processing conditions evaluated in spray-drying (SD), extended storage and UV processing steps.

Input	Value/Distribution
SD outlet particle temperature (°C)	*~Uniform* (82.7, 85.5)
SD particle residence time (s)	*~Uniform* (30, 60)
UV dosage (J/L)	*~Normal* (3,251, 65)
Extended storage time at 22°C (d)	14

The literature review provided data on the effect of spray-drying on Porcine epidemic diarrhea virus (PEDV) and African swine fever virus (ASFV) in porcine plasma with reduction levels ranging from 4.0 to 5.0 log (Supplementary Table S1) (6, 18–20). The effect of SDPP storage at room temperature (20°C) for 14 days has also been evaluated using PEDV and ASFV with inactivation levels ranging from 3.5 to 7.0 log (Supplementary Table S2) (19–23). Data on the effects of UV processing in raw porcine plasma were obtained from published studies and used to estimate the D-values (energy in J/L required to reduce 1 log or 90% of the initial virus concentration) of PEDV and ASFV with values ~490 J/L (Supplementary Table S3) (5, 6).

### Performance objective (PO) levels in spray-dried porcine plasma

The PO concept applied to viruses in feed is related to the contamination of the raw ingredient and inactivation achieved during the individual or multiple CCP used in the production process, and was calculated using the following equation (16):


(1) $$H_{0}-\sum R+I\leq PO$$

Where *H*_0_ is the contamination level of viruses in raw plasma (log HAD_50_ or TCID_50_/mL), the sum of R is the reduction level (log) achieved in all critical control points combined, and *I* is the increase in virus concentration due to water removal (plasma concentration). To estimate a generic PO level in the manufacturing of SDPP that can be applied to any swine virus, information about SDPP lot sizes was obtained from the NASDBPP as 10, 15, and 20 metric tons. Absence of any infectious viral particle in 1.0 × 10^7^, 1.5 × 10^7^ and 2.0 × 10^7^ g of SDPP will result in less than 1.0 × 10^−7^, 6.7 × 10^−8^, and 5.0 × 10^−8^ viral particles/g (less than 1 viral particle in 1.0 × 10^7^ g for a final concentration of 1×10^−7^ viral particles/g for the 10 metric ton example) analogous to a final concentration in log scale in plasma of −7.0, −7.2, and −7.3 log particles/g. These are the PO levels needed to produce a virus-free lot of plasma (less than one infectious particle in the entire lot) based on the different lot sizes produced.

To estimate the total log reduction needed (R) to achieve an adequate PO level for SDPP, the concentration of infectious swine viruses in raw plasma was estimated from the raw material used to manufacture SDPP. Data on viral concentration of PEDV in SDPP were published from monthly collected samples during an entire year in EU, Brazil, and United States processing plants (24). Using a conservative approach, it was assumed that TCID_50_ values derived through a calibration curve from a qPCR corresponded to infective viral particles in SDPP in the model (24).

### ASFV load in raw plasma during a pre-clinical scenario

The worst-case scenario in any animal disease outbreak occurs when viremic pigs (infected pigs that are shedding virus) go unnoticed during antemortem inspections and enter the food or feed chain. During this pre-clinical scenario, blood may be collected from viremic asymptomatic pigs. However, the concentration of ASFV in the blood collected under a pre-clinical scenario is unknown. A literature search was performed to identify studies that quantified the ASFV load in blood collected from naturally infected pigs during an ASF outbreak using different virus strains (pigs naturally infected from artificially inoculated pigs) from Georgia, Armenia, Poland, Estonia, Latvia, Poland, China, and Russia (25–30). Supplementary Table S4 shows the modeling approach used to simulate the time to the appearance of clinical signs (assumed same as detection of clinical signs) and slaughtering time up to the detection of clinical signs in each of the studies to simulate the ASFV load in the blood of pre-clinical pigs.

A probabilistic model was developed to simulate the mean ASFV concentration in blood and raw plasma of viremic animals at different time intervals during an ASF outbreak by characterizing the time to observe clinical signs (Tc) and the time blood from pigs would be collected before the detection of clinical signs (Stdb). The ASFV concentration in blood was simulated at different time intervals by IF statements depending on when the blood was collected before symptom detection (Supplementary Table S4). All ASFV values simulated in the model were assumed to be infective.

### Monte Carlo simulation

Probabilistic models were developed using Microsoft Excel and @Risk 7.6 (Palisade Corp., NY) to estimate the PO levels under different batch size scenarios, log inactivation by different processing scenarios, and ASFV concentration in raw plasma. Each model input variable was characterized by its inherent variability (i.e., industrial processing conditions, standard deviation of virus concentration between SDPP batches, virus inactivation rates achieved in each processing technology) and uncertainty using a probability distribution. Model outputs were obtained by numerical simulation techniques (100,000 iterations to assure convergence). For each iteration, a Latin Hypercube sampling technique was used to draw one random value of each variable or parameter from its respective distribution, creating simulated populations that represented the model outcomes. Output estimates were characterized by the mean and 95% confidence interval (CI) values.

### PO compliance rates

Compliance rates (%) expressed as the probability that a PO level was achieved were estimated for PEDV and ASFV by using the ICMSF control measures validation (PO) tool (downloaded from the ICMSF website) and Equation 1 (16). Mean and standard deviation values were imputed into the tool for the initial PEDV and ASFV concentration in raw liquid plasma (H_0_), log reduction of each processing stage (R1-spray-drying, R2-UV and R3-extended storage) and increase in virus concentration due to water removal in plasma (concentrating the liquid plasma from 8.5 to 30% solids for a 3.5 concentration factor or 0.5 log increase in virus concentration).

## Results and discussion

### PO level in SDPP

Traditionally, the main challenges with developing a Food and Feed Safety Objective (FSO) and related regulatory limits for viruses in food or feed are that there are no internationally accepted microbiological limits, there are numerous swine viruses, the uncertainty related to the oral dose required to produce infection in a large animal population, and the limited data availability related to the viral load of swine viruses in raw materials and feed ingredients. For these reasons, this study was focused on establishing a generic PO level at the end of SDPP manufacturing process that can be applied to any swine virus and the production of 10-, 15-, and 20-tons batches of SDPP that are virus-free.

### Virus inactivation under different processing scenarios

The extent of heat inactivation of viruses varies depending on virus type and strain, virus titer, substrate matrix characteristics (pH, fat and protein content, ionic strength), and heat application (wet vs. dry). The effect of heat treatment on animal viruses has been studied for the last 50 years, and thus, extensive research and knowledge are available on this topic. The main heat inactivation mechanisms observed for viruses are the damage/disintegration of the capsid protein, degradation/release of nucleic acid, and destruction of receptor binding (31).

The baseline processing scenario used by porcine plasma manufacturers in the EU and US involves a combination of spray-drying and extended storage according to the conditions in Table 1. Drying inactivation kinetics can be summarized in two main events: dehydration and dry-heat inactivation. The effectiveness of dehydration depends on the inlet air temperature (T), solids content, and particle size whereas the dry-heat inactivation kinetics are affected by the outlet air T and residence time in the dryer (32, 33). Both CCPs rely on a combination of dehydration and dry-heat phenomena to reduce virus infectivity. Results from published studies (6, 20, 22, 34) have shown that spray drying in addition to extended storage (i.e., SD + extended storage) are capable of inactivating a wide range of naked and enveloped RNA and DNA swine viruses. The risk assessment model showed mean inactivation levels of PEDV and ASFV ranged from 8.4 to 11.1 log (Table 2).

**Table 2:** Effect of spray-drying, UV, and extended storage on the inactivation of PEDV and ASFV by using the Monte Carlo simulation.

Virus conventional name	Inactivation level (log reduction ± SD)					
	Spray-drying (SD)	Storage	UV	Combined SD + storage^*^	Combined SD + UV + storage^*^	References
PEDV	5.1 ± 0.2	3.2 ± 0.8	6.6 ± 0.1	8.3 (7.9–8.7)	15.0 (14.7–15.1)	(5, 19, 20)
ASFV	4.1 ± 0.2	7.0^**^	6.8 ± 0.1	11.1 (10.7–11.5)	17.9 (17.4–18.3)	(6, 22)

^*^Mean and 95% CI after Monte Carlo simulation with 100,000 iterations. ^**^D-value of 2-days simulating a storage of 14-days at 22°C (22).

There is extensive research showing that UV-C is capable of significantly inactivating most human and animal viruses. The effectiveness of UV-C depends mainly on the liquid absorbance, which requires greater energy as the absorbance increases. Risk assessment model showed virus inactivation levels after UV processing >6.5 log of PEDV and ASFV (Table 2). Research results have shown that UV light induces reactive oxygen species that may interact with the lipid membrane of enveloped viruses causing lipid peroxidation (5). Current international guidelines by WHO for human blood plasma products require a 4.0 log reduction for viruses of concern (35). The U.S. Food and Drug Administration (FDA) guidelines for pasteurization of fruit juices using non-thermal technologies such as UV, requires a 5.0 log reduction of the pathogen of concern (36, 37). The UV processing conditions used by SDPP manufacturers that have adopted this technology in the US and EU would meet these requirements for PEDV and ASFV. The combination of baseline + UV provides an additional inactivation level that exceeds 15.0 log (Table 2).

### PEDV and ASFV concentration in raw plasma during a pre-clinical scenario (H_0_)

The PEDV virus load from SDPP samples collected were measured as TCID_50_ estimated from qPCR values using standard calibration and were assumed to indicate infectious viral particles (24). Viral load in liquid plasma was back calculated to assume 48 g of dried plasma was produced from 600 g of liquid plasma (12.5 dilution factor). Mean PEDV concentration in SDPP was estimated as 0.1 ± 0.6 log TCID_50_/g whereas the concentration of PEDV in liquid plasma was estimated as −1.0 ± 0.6 log TCID_50_/mL (mean ± SD) (H_0_).

The ASFV concentration in blood during natural infection varies among studies conducted, where the time for pigs to show clinical signs after exposure ranged from 4-to 18-days post-infection (dpi), and ASFV was found in blood after 6–7 dpi reaching maximum values (10^6^–10^9^ HAD_50_/mL) after 9–16 dpi (Supplementary Table S4). The ASFV concentration in the blood of viremic undetected pigs can occur before the detection of clinical signs (signs) in a relatively short period. The mean concentration of ASFV during a pre-clinical scenario was estimated as the arithmetic mean of ASFV concentration values from all studies to be 0.6 (0.1–1.4) log HAD_50_/mL (H_0_). A European Food Safety Authority (EFSA) scientific opinion regarding the risk of ASFV entering the EU through feed and bedding materials, ranked spray-dried blood plasma as a low-risk feed matrix due the fact that there is a short period in which animals can be infected without showing clinical signs, combined with the production procedures for the SDPP products in the EU that avoid the collection of plasma from ASF-infected areas (17).

In our current model, we assumed the time to detect ASF was equal to the appearance of clinical signs and thus after that time pigs would become clinical, and blood not collected. This may vary depending on the extent of active surveillance of clinical signs by swine producers and/or the virulence of the ASFV variant, where the disease transmission rate and the onset of clinical signs will greatly vary (38). Previous studies using Foot and Mouth Disease (FMD) outbreak simulations in the U.S. have shown that outbreak detection may be delayed due to the lack of expertise to detect clinical signs by visual inspection and delays in official testing to detect the disease (39). The extent of delay in outbreak detection for ASFV is unknown. This potential scenario may increase the time pigs would enter the feed chain and increase the ASFV concentration in the collected blood. The EFSA (17) report also indicated that as ASFV continues to spread, there is an increased risk that preclinical viremic animals might go undetected at ante-and post-mortem inspection in slaughterhouses from recently ASFV-infected areas where official surveillance has not implemented yet (17).

### PO compliance rates in SDPP

The PO compliance rates (% of lots that comply with the PO level) for PEDV and ASFV at different batch sizes and processing scenarios are shown in Tables 3, 4 by using ICMSF equation 1. Baseline processing scenario compliance rates were 97.0–98.7% for PEDV and 100% for ASFV due to the effectiveness of spray-drying and extended storage on virus inactivation. Recent results reported by Fischer et al. (22) showed an estimated *D*-value of 2.0 days for ASFV during storage of SDPP at room temperature (21 ± 2°C), which represented a 7.0 log reduction after 2 weeks of storage. However, studies performed on the survival of viruses in feed during storage need further validation because the effect of the chemical and physical composition of feed matrices (i.e., lipid, protein, and moisture content, and water activity) is still unknown. Experimental studies on ASFV survival in different feed ingredients simulating the storage conditions of a transoceanic shipment estimated half-time values (time to reduce 50% of the initial virus concentration) of 4.1–5.1 days (calculated *D*-value of 13.6 to 16.9 days) (40) and 7.7 to 15.9 days (calculated *D*-value of 25.6–52.8 days) (41). These values represent a much longer survival period (more than 10× higher in some cases) than the previously stated values for SDPP. The experimental procedure used to inoculate viruses into feed to evaluate virus survival during storage conditions significantly increases the moisture content (water activity) of the matrix, which may have an impact on virus survival. This may explain the differences observed in ASFV survival among studies where moisture content varies depending on the initial inoculation procedure and the storage conditions (temperature and ambient moisture), which change the rate of sample drying and thus, virus survival.

**Table 3:** Percentage of compliance with the PO levels at various processing and lot sizes scenarios for PEDV by using the ICSMF equation.

Ho (viral load liquid plasma)	Mean concentration (log TCID_50_/g)	Sd
	−1.0	0.6
Processing step	Mean inactivation (log TCID_50_/g)	Sd
Ultraviolet	−6.6	0.1
Increase due to plasma concentration^*^	0.5	–
Spray-Drying	−5.1	0.2
Extended storage	−3.2	0.8
Final concentration SDPP (log TCID_50_/g)	−8.7 ± 1.0	−15.3 ± 1.0

	SD + storage	SD + UV + storage
Probability PO 10 mt batch (−7.0 log) (%)	95.7	100
Probability PO 15 mt batch (−7.2 log) (%)	93.6	100
Probability PO 20 mt batch (−7.3 log) (%)	92.2	100

^*^Assuming solids concentration from 8 to 30% (3.5 concentration factor).

**Table 4:** Percentage of compliance with the PO levels at various processing and lot sizes scenarios for ASFV by using the ICMSF equation.

Ho (viral load plasma)	Mean concentration (log TCID_50_/g)	Sd
	0.6	0.3
Processing step	Mean inactivation (log TCID_50_/g)	Sd
Ultraviolet	−6.8	0.1
Increase due to plasma concentration^*^	0.5	–
Spray-drying	−4.1	0.2
Extended storage	−7.0	0.8^**^
Final concentration SDPP (log TCID_50_/g)	−10.0 ± 0.9	−16.8 ± 0.9

	SD + storage	SD + UV + storage
Probability PO 10 mt batch (−7.0 log) (%)	100	100
Probability PO 15 mt batch (−7.2 log) (%)	100	100
Probability PO 20 mt batch (−7.3 log) (%)	100	100

^*^Assuming solids concentration from 8 to 30% (3.5 concentration factor). ^**^When sd was not estimated in the original study, a default value of 0.8 was used based on the natural microbial variability (14).

Another variable that is often overlooked in cell culture-based assays is the impact of the substrate matrix on the genetic or physiological characteristics of the cells. In most ASFV inactivation studies performed in feed during storage, the feed matrix may alter the ability of the cell culture to become either infected, lose its ability to amplify, or release progeny making the inactivation data reported in the studies questionable because the observed ASFV infectivity loss may only be transient. As a result, these uncertainties could impact the PO compliance assessments for ASFV in SDPP.

It is important to recognize that SDPP lots that are not in compliance with the stated PO levels does not imply that those lots would have enough virus particles to produce infection. The true meaning of the PO levels established in this study is the absence of any infectious virus particles in the entire lot without taking into effect subsequent conditions that may lead to animal infection such as the oral infective dose that may range from 56 TCID_50_ in case of PEDV to 10^4^ TCID_50_ for ASFV, respectively (42, 43). In addition, the inactivation levels observed in previous studies when raw plasma was inoculated with these viruses may be less than the actual reduction level due to constraints in the amount of initial virus titer that was used in the experiments (34).

The PO compliance rates of the baseline + UV processing scenario increased the inactivation levels, providing a 100% compliance rate for all scenarios. As previously discussed, the combination of several inactivation factors (rapid dehydration, dry heat, UV, and low moisture storage), known as hurdle technology, greatly improves the effectiveness of these processing scenarios for achieving adequate biosecurity to prevent infection of pigs on farms.

The viral concentration estimates used in this assessment do not include the potential for virus recontamination of processed products. Re-contamination has been recognized as a significant component of the risk of ASFV introduction into the U.S. via contaminated corn and soybean meal (44). Future risk assessment studies should focus on the development and optimization procedures that minimize the risk of virus recontamination of processed SDPP (45).

## Conclusion

Spray-dried porcine plasma production processes include a variety of critical control points and inactivation mechanisms that provide significant inactivation levels (8.4–11.1 log reduction) for a wide range of naked and enveloped RNA and DNA swine viruses. Under the current conditions, our model estimated that an inactivation level (log-kill) of 7.0, 7.2, and 7.3 logs must be achieved to manufacture an infectious-free SDPP batch of 10, 15, and 20 metric tons, respectively. The PO compliance rates were >95% for the baseline SDPP production scenario and 100% for the baseline + UV processing scenario. Future research on the survival of viruses in more realistic storage conditions is needed to understand the chemical and environmental factors affecting viral inactivation, and to validate or refute the inactivation effects reported from previous studies in the literature.

## Data availability statement

The original contributions presented in the study are included in the article/Supplementary material, further inquiries can be directed to the corresponding author.

## Author contributions

FS: Conceptualization, Data curation, Formal analysis, Investigation, Methodology, Visualization, Writing – original draft, Writing – review & editing. PU: Conceptualization, Funding acquisition, Supervision, Writing – review & editing. JL: Funding acquisition, Project administration, Writing – review & editing. DS: Funding acquisition, Writing – review & editing. GS: Funding acquisition, Project administration, Supervision, Writing – review & editing.
