# Supplementary material for: Quantitative risk assessment model of the presence of porcine epidemic diarrhea and African swine fever viruses in spray-dried porcine plasma
Source: Front Vet Sci. 2024 Jun 12;11:1371774. doi: 10.3389/fvets.2024.1371774 (PMC11202310; doi:10.3389/fvets.2024.1371774)
Supplement: Supplementary file 1 [file Table_1.DOCX]

Table S1: Effect of spray-drying on the survival of PEDV and ASFV in spray dried porcine plasma.

| **Virus name^1^** | **Inactivation after spray-drying** | **Reference** |
| --- | --- | --- |
| PEDV | 4.2 log  Bench-top at inlet T of 166°C and outlet T of 80°C for <1 s | Gerber et al., 2014 |
|  | 5.1±0.2 log*  Bench-top at inlet T of 200°C and outlet T of 80°C for <1 s + 80°C for 30 s | Pujols and Segales, 2014 |
|  | 1.4 log  Bench-top at inlet T of 190°C and outlet T of 80°C for <1 s | Hulst et al., 2019 |
| ASF | 4.1±0.2 log*  Bench-top at outlet T of 80°C for <1 s + 80°C for 60 s | Blázquez et al., 2021 |

*: These studies were performed at a combination of spray-drying and dry-heat for 30 and 90 s at 80^o^C. ^1^Porcine epidemic diarrhea virus (PEDV), African swine fever virus (ASFV).

Table S2: Effect of heated storage (20±2°C for 14 days) on PEDV and ASFV survival in spray dried porcine plasma.

| **Virus** | **Log reduction or D-value (1 log reduction)** | **Reference** |
| --- | --- | --- |
| PEDV | D25°C: 1.1±1.2 days | Trudeau et al., 2017 |
|  | 4-5 log after 1 day at freezing outdoor conditions (-10 to 0°C) | Dee et al., 2015 |
|  | 2.6 log | Hulst et al., 2019 |
|  | 3.8 log | Pujols and Segales, 2014 |
| ASFV | >5.7 log (D25°C: 2 days) | Fisher et al., 2021 |

Table S3: Effect of UV-C continuous processing against PEDV and ASFV in porcine plasma by using a continuous flow SurePure (Milnertown, South Africa) ultraviolet (UV) system at 22.95 J/L per cycle.

| **Virus** | **D-value (UV energy to reduce 1 log) (J/L)** | **Reference** |
| --- | --- | --- |
| PEDV | 488.2 J/L | Blázquez et al., 2019a |
| ASFV | 478.1 J/L | Blázquez et al., 2021 |

Table S4: Input parameters to estimate the ASFV load in blood from naturally infected pigs.

| **Time to clinical signs (Tc) (days)*** | **Slaughter time before detection (Stbd) (days)** | **Mean ASFV load in blood during infection (log HAD_50_/mL)***** | **# pigs** | **Dose (HAD_50_/mL)** | **ASFV strain** | **Reference** |
| --- | --- | --- | --- | --- | --- | --- |
| *~Normal* (9.9, 1.6)** | Uniform (0, Tc) | IF(Stdb<=7,0,IF(AND(Stdb>7,Stdb<=8),1, IF(AND(Stdb>8,Stdb<=9),3,6.5))) | 16 | 100 | Georgia | Guinat et al., 2014 |
| *~Normal* (12.7, 2.0) |  | IF(Stdb<=7,0,IF(AND(Stdb>7,Stdb<=8),0.5, IF(AND(Stdb>8,Stdb<=9),0.7, IF(AND(Stdb>9,Stdb<=10),1.5, IF(AND(Stdb>10,Stdb<=11),2,2.5))))) | 8 |  |  |  |
| 11.0 |  | IF(Stdb<ASFVb,0,Uniform(0,6)) | 4 | 1,000 | Armenia | Yamada et al., 2020 |
| 11.0 |  | IF(Stdb<ASFVb,0,Uniform(0,5.5)) |  |  |  |  |
| 10.0 |  | IF(Stdb<ASFVb,0,Uniform(0,5)) |  |  |  |  |
| 11.0 |  | IF(Stdb<ASFVb,0,Uniform(0,3)) |  |  |  |  |
| *~Normal* (9.0, 1.0) |  | IF(Stdb <7,0,IF(AND(Stdb >=7, Stdb <10),3.5, IF(AND(Stdb >=10, Stdb <13),6, IF(AND(Stdb >=13, Stdb <16),7.9,6)))) | 4 | 10 | Poland | Gallardo et al., 2021 |
| *~Normal* (13.0, 0.0) |  | IF(Stdb <7,0,IF(AND(Stdb >=7, Stdb <10),0.5, IF(AND(Stdb >=10, Stdb <13),8.8, IF(AND(Stdb >=13, Stdb <16),7,7)))) | 4 |  | Estonia |  |
| *~Normal* (11.5, 0.0) |  | IF(Stdb <7,0,IF(AND(Stdb >=7, Stdb <10),1, IF(AND(Stdb >=10, Stdb <13),7, IF(AND(Stdb >=13, Stdb <16),5,5)))) | 4 |  | Latvia |  |
| *~Normal* (8.3, 1.5) |  | IF(Stdb <=6,0, IF(AND(Stdb >6, Stdb <=7),4,IF(AND(Stdb >7, Stdb <=9),8.5,9))) | 7 | 10,000 | Poland | Olesen et al.,2017 |
|  |  | IF(Stdb <=6,0,IF(AND(Stdb >6, Stdb <=7),4,IF(AND(Stdb >7, Stdb <=9),8.5,9))) |  |  |  |  |
|  |  | IF(Stdb <=7,0,IF(AND(Stdb >7, Stdb <=9),8.5, IF(AND(Stdb >9, Stdb <=11),8.5,9))) |  |  |  |  |
|  |  | IF(Stdb <=8,0,IF(AND(Stdb >8, Stdb <=9),2.5, IF(AND(Stdb >9, Stdb <=10),7,9))) |  |  |  |  |
|  |  | IF(Stdb <=9,0,IF(AND(Stdb >9, Stdb <=10),5.5, IF(AND(Stdb >10, Stdb <=11),8,9))) |  |  |  |  |
|  |  | IF(Stdb <=10,0,IF(AND(Stdb >10, Stdb <=11),3, IF(AND(Stdb >11, Stdb <=14),8,9))) |  |  |  |  |
|  |  | IF(Stdb <=10,0,IF(AND(Stdb >10, Stdb <=11),3,IF(AND(Stdb >11, Stdb <=14),8,9))) |  |  |  |  |
| *~Normal* (10.0, 0.8) |  | IF(Stdb <=6,0,IF(AND(Stdb >6, Stdb <=7),2.5,IF(AND(Stdb >7, Stdb <=9),8,9))) | 7 |  |  |  |
|  |  | IF(Stdb <=7,0,IF(AND(Stdb >7, Stdb <=9),7.5,IF(AND(Stdb >9, Stdb <=11),9,9))) |  |  |  |  |
|  |  | IF(Stdb <=7,0,IF(AND(Stdb >7, Stdb <=9),4, IF(AND(Stdb >9, Stdb <=11),8.5,9))) |  |  |  |  |
|  |  | IF(Stdb <=9,0,IF(AND(Stdb >9, Stdb <=10),3,IF(AND(Stdb >10, Stdb <=11),6.5,9))) |  |  |  |  |
|  |  | IF(Stdb <=10,0,IF(AND(Stdb >10, Stdb <=11),4,IF(AND(Stdb >11, Stdb <=14),9,9))) |  |  |  |  |
|  |  | IF(Stdb <=11,0,IF(AND(Stdb >11, Stdb <=12),6.5,IF(AND(Stdb >12, Stdb <=14),9,9))) |  |  |  |  |
|  |  | IF(Stdb <=15,0,6) |  |  |  |  |
| 9.0 |  | IF(Stdb <Tc,0, Uniform(7.5,8)) | 2 | 31,623 | China | Zhao et al., 2019 |
| 9.0 |  | IF(Stdb <Tc,0, Uniform(7.5,8)) |  | 3162 |  |  |
| *~Uniform* (4.0, 9.0) |  | IF(Stdb <Tc,0, Uniform(3.5,6.5)) | 4 | 1000 | Russia | Vlasov et al., 2020 |

*: It is assumed that time to detect the disease equals to the time clinical signs appeared. **: The normal distribution was truncated at 0 at the minimum value. ***: It is assumed that HAD_50_/mL values obtained by qPCR are infective. ND: Not determined.
